# Effects of Mixed Decomposition of *Pinus sylvestris* var. *mongolica* and *Morus alba* Litter on Microbial Diversity

**DOI:** 10.3390/microorganisms10061117

**Published:** 2022-05-28

**Authors:** Jiaying Liu, Yawei Wei, You Yin, Keye Zhu, Yuting Liu, Hui Ding, Jiawei Lei, Wenxu Zhu, Yongbin Zhou

**Affiliations:** 1 College of Forestry, Shenyang Agricultural University, Shenyang 110866, China; ljying222@163.com (J.L.); 2013500010@syau.edu.cn (Y.W.); 1993500012@syau.edu.cn (Y.Y.); ansizky@163.com (K.Z.); lyt_edu@163.com (Y.L.); a15502410421@163.com (H.D.); leijiwei123456@163.com (J.L.); 2 Institute of Modern Agricultural Research, Dalian University, Dalian 116622, China; 3 Research Station of Liaohe-River Plain Forest Ecosystem, Chinese Forest Ecosystem Research Network (CFERN), Shenyang Agricultural University, Tieling 112000, China; 4 Life Science and Technology College, Dalian University, Dalian 116622, China

**Keywords:** litter decomposition, microbial community, *Pinus sylvestris* var. *mongolica*, *Morus alba*

## Abstract

*Pinus sylvestris* var. *mongolica* is widely planted in China as a windbreak and sand fixation tree. To improve the current situation of large-scale declines of forested areas planted as *P. sylvestris* var. *mongolica* monocultures, we investigated the biological and microbial effects of stand establishment using mixed tree species. The interactions during the mixed decomposition of the litter and leaves of different tree species are an important indicator in determining the relationships among species. In this experiment, a method of simulating the mixed decomposition of *P. sylvestris* var. *mongolica* and *Morus alba* litter under *P. sylvestris* var. *mongolica* forest was used to determine the total C, total N, and total P contents in the leaf litter, and the microbial structures were determined by using Illumina MiSeq high-throughput sequencing. It was found that with samples with different proportions of *P. sylvestris* var. *mongolica* and *M. alba* litters, the decomposition rate of *P. sylvestris* var. *mongolica* × *M. alba* litter was significantly higher than that of the pure *P. sylvestris* var. *mongolica* forest, and the microbial community and composition diversity of litter in a pure *P. sylvestris* var. *mongolica* forest could be significantly improved. The possibility of using *M. alba* as a mixed tree species to address the declines of pure *P. sylvestris* var. *mongolica* forest was verified to provide guidance for pure *P. sylvestris* var. *mongolica* forests by introducing tree species with coordinated interspecific relationships and creating a mixed forest.

## 1. Introduction

*Pinus sylvestris* var. *mongolica* is naturally distributed in the northern mountains of the Greater Khingan Mountains and the Hulun Buir Sandy Steppe in China. It has excellent characteristics such as cold resistance, drought resistance, barren resistance, and rapid growth [1]. It is the main tree species used for creating building shelter forests, soil and water conservation forests, and timber forests [2]. Since the 1950s, it has been successfully introduced and cultivated in 13 provinces (autonomous regions) in China and has been introduced and planted on a large scale as one of the main afforestation tree species in the “Three-North” Shelter Forest Program. It significantly improved the soil nutrient (e.g., carbon, nitrogen, and phosphorus) conditions [3,4], water conditions [5], microbial community compositions [6,7], and enzyme activities [8] in afforestation land. In 1978, the State Council of China carried out the construction of the “Three-North” Shelter Forest Program to solve the contradiction between the backward forestry productivity in China’s three-north regions (e.g., Northwest, Northeast, and North China) and the society’s growing ecological, material and cultural demands for forestry. Since the beginning of the “Three-North” Shelter Forest Program in China, large numbers of artificial pure pine forests have been planted in the arid and semiarid regions of North China, Northeast China, and Northwest China [9]. At present, the total area of artificial pure *P. sylvestris* var. *mongolica* forest in China has reached 3000 km^2^, which has provided very significant economic, social and ecological benefits [10]. Therefore, the success or failure of *P. sylvestris* var. *mongolica* plantations is an important indicator of the success of the “Three-North” Shelter Forest Program.

However, since the early 1990s, the earliest introduced *P. sylvestris* var. *mongolica* plantations (located at the southern edge of Horqin Sandy Land, Zhanggutai area) had yellow branches, weak growth, the occurrence of pests and diseases, and whole plants then died and could not be regenerated naturally [11,12,13,14,15]. After that, similar situations occurred in Shaanxi, Shanxi, Heilongjiang, Jilin, and other provinces. Among them, 65% of the *P. sylvestris* var. *mongolica* plantation in the sandy land of nearly 383 km^2^ in Liaoning Province declined [16]. Nearly 40% of the existing 200 km^2^ *P. sylvestris* var. *mongolica* sand-fixing forests in Horqin Sandy Land were in a state of decline [17]. These problems seriously affect the stability and sustainability of forest ecosystems.

Compared with pure forests, mixed forests can significantly improve the plant utilization efficiencies of soil nutrients and water, increase the effectiveness of resource space utilization and biodiversity, and have more advantages in enhancing stand stress resistance and stability [18,19]. Therefore, creating a mixed forest of *P. sylvestris* var. *mongolica* with other suitable tree species can be an effective way to address the problem of forest stand growth. The key to the success of mixed afforestation is whether the relationships among the species of quasi-mixed trees are coordinated [20], that is, whether they are conducive to the sustainable development of forestland. To this end, it is necessary to study the interspecific relationships between *P. sylvestris* var. *mongolica* and common tree species.

As an excellent native tree species in China, *Morus alba* has a wide geographical distribution, strong adaptability, high afforestation survival rate, and large canopy and can be used for ecological afforestation. At present, many researchers have found that *M. alba* has strong resistance to a variety of adverse site environments, and it has excellent salt and alkali resistance, barren resistance, drought resistance, and cold resistance [21]. Therefore, it can play the role of regulating the climate and ecology, such as maintaining water and soil, conserving water sources, and purifying the air in places with fragile ecological environments [22]. In addition, because *M. alba* grows quickly, when it is planted in a sandy wasteland, it can effectively improve forest coverage in windy and sandy areas. Therefore, *M. alba* can use its ecological advantages to provide full play to its strengths in soil and water conservation, desertification control, returning land for farming to forestry, and other aspects in the sandstorm area in northwestern Liaoning, where the ecological environment is fragile and has become an excellent tree species for ecological management [23,24] and can thereby stabilize the ecological functions and environments in sandy areas. At present, *M. alba*, as one of the important ecological tree species in ecological environment construction, has played an important role in the control of desertification, rocky desertification, and sandy land in Xinjiang, Shaanxi Loess Plateau, Chongqing, Guangxi, and Beijing [25].

As an intermediate carrier for plant nutrient return, forest litter is the main supplier of forest soil self-fertilization [26] and plays a bridge role in the nutrient cycles of forest ecosystems [27]. The interactions of mixed decomposition of litters from different tree species during decomposition have become an important indicator to measure interspecific relationships [28], which will directly affect litter decomposition, nutrient release, soil nutrient balance, and enzyme activity [29], which thus affect the nutrient cycles of mixed forest ecosystems [30]. A large number of studies have proven that the mixed decomposition of litter and leaves exhibits complex nonadditive effects [31,32] and that mixed decomposition of different tree species results in different decompositions and releases of different nutrient components [33]. The litter of coniferous species and broad-leaved tree species have different decomposition rates due to the differences in their substrates. Generally, the litter of broad-leaved tree species decomposes faster due to the high ash content [34,35]. When conifer species and broad-leaved species are mixed, the amount and composition of litter leaves are changed, which results in accelerated decomposition and nutrient release, which in turn affects the nutrient cycles and soil nutrient accumulations in woodlands and then improves the soil nutrient contents and soil fertility.

In summary, studying the decomposition characteristics of mixed litter can reveal the interactions among *P. sylvestris* var. *mongolica* and common tree species in the mixed decomposition process of litter and leaves and can provide a basis for exploring the relationships among tree species. Such research can provide guidance for the introduction of tree species with coordinated interspecific relationships and the creation of mixed forests in pure forests of *P. sylvestris* var. *mongolica*. By studying the mixed decomposition characteristics of different tree species and their effects on the soil’s physicochemical and biological properties, analyzing the interspecific relationships of tree species has become a research hotspot and can then be used to provide suggestions for the construction of mixed forests. A large number of studies have proven that mixing *P. sylvestris* var. *mongolica* and broad-leaved tree species can significantly enhance soil fertility, increase stand growth [36], improve soil microbial contents [37], and reduce forest mortality and pest rates [38]. Based on the above experimental results, to address the scientific problem of using *M. alba* × *P. sylvestris* var. *mongolica* mixed forests to ameliorate the decline of *P. sylvestris* var. *mongolica* forests, the following scientific hypotheses are put forward: (1) *M. alba* × *P. sylvestris* var. *mongolica* mixed forests can improve the physical and chemical properties of leaves. (2) Compared with pure *P. sylvestris* var. *mongolica* forests, mixed forests of *M. alba* and *P. sylvestris* var. *mongolica* can significantly improve the diversity of the leaf microbial community. This study provides a theoretical basis for the construction of mixed *M. alba* × *P. sylvestris* var. *mongolica* forests in sandy land.

## 2. Materials and Methods

### 2.1. Site Description

**Figure 1:**
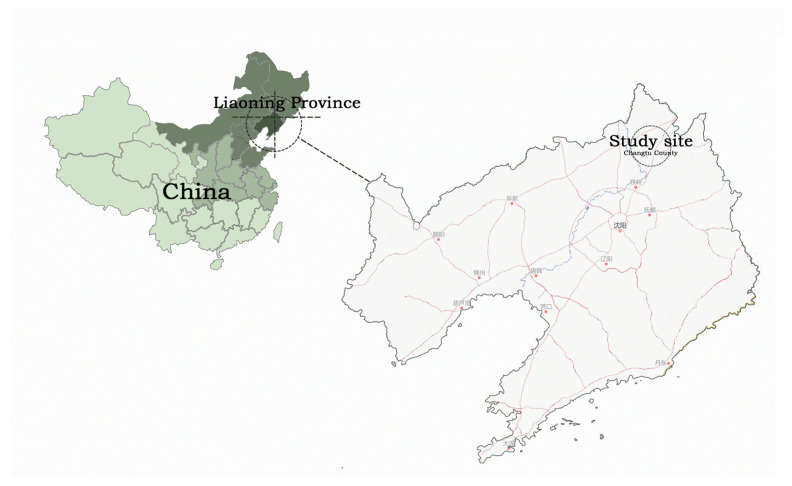
Geographical location of the study site.

The study site was established at the Liaohe Plain Forest Ecological Station of the State Forestry and Grassland Administration (Fujia Machinery Forest Farm, Changtu County, Tieling City, Liaoning Province) (43°21′143″–42°53′623″ N, 123°53′623″–123°53′623″ E), which is located on the southeastern edge of Horqin Sandy Land, where three provinces, Liaoning, Jilin and Inner Mongolia meet. The landform consists of the Liaohe alluvial plain type, with an elevation of 91.10–173.40 m. It has a temperate semihumid and semiarid continental climate with an average annual precipitation level of 400–550 mm, which is mostly concentrated in July and August each year, with annual evaporation of 1843 mm, extreme maximum temperature of 35.6 °C, extreme minimum temperature of −31.5 °C, and daily average temperature of 6.3 °C. The soil type is yermic with low contents of organic matter and other nutrients. The main vegetation community in this area consists of a windbreak and sand-fixation forest with *P. sylvestris* var. *mongolica* as the main component, with a planting area of 54.86 km^2^ and canopy closure of 0.7. The terrain is flat, and the understory contains a few shrubs and herbs (Figure 1).

### 2.2. Litter Sampling

Fresh *P. sylvestris* var. *mongolica* and *M. alba* litters were collected in the middle of October 2020 when they began to shed their leaves, and homemade 1 m × 1 m litter collectors (Figure S1) were set up in the study area to collect freshly fallen leaf samples over a 10-day period. The litter samples were then mixed and stored in an ice box for immediate return to the laboratory. The collected litter samples were placed into nylon mesh decomposition bags with sizes of 0.2 m × 0.2 m (aperture of 1 mm × 1 mm). The total mass of each bag was 8 g, and the mass ratios of the *P. sylvestris* var. *mongolica* and *M. alba* were 0:1, 1:1, and 1:0 (respectively recorded as Ma, PsMa, and Ps, respectively). In each plot, 8 decomposing bags with different proportions of litter and leaves were set up, and 4 plots were repeated. The four plots were arranged in the same *P. sylvestris* var. *mongolica* forest, and the distance between each plot was greater than 50 m. In April 2021, a mixed decomposition test was set up under the *P. sylvestris* var. *mongolica* forest. We retrieved the decomposition bags in July 2021, carefully removed the soil or debris on the surfaces of the litter decomposition bags, stored them in an ice box, and immediately transported these bags back to the laboratory.

Then, the 8 decomposing bags of fallen leaves from the same plot were mixed evenly, weighed, and divided into 2 parts. One part of the litter was dried at 65 °C to constant weight. The dried litter samples were crushed and pulverized through a 0.15 mm sieve (100 mesh). Their chemical properties were determined, including the total carbon, total nitrogen, and total phosphorus contents. The second part was stored at −80 °C for molecular biology determinations.

### 2.3. Determination of Litter Characteristics

The total nitrogen (Total N) and total carbon (Total C) contents of leaves were determined with an elemental analyzer (Elementar Vario EL III, Hesse, Germany) [39]. The total phosphorus (Total P) contents were determined by using the molybdenum-antimony anti-spectrophotometric method [40].

### 2.4. DNA Extraction and Amplification Sequencing

The second part of the litter samples was immediately processed for DNA extraction. In each leaf replicate, 30 g of litter specimens was placed in a 1000 mL sterile Erlenmeyer flask, and 500 mL of sterile PBS buffer (pH 7.4, 1× phosphate buffered saline) was then added. In order to wash the microbial cells on the leaves, sonication was performed in an ultrasonic cleaning bath at a frequency of 40 kHz for 6 min, shaking at 200 r/min for 20 min at 30 °C, and then sonication (frequency 40 kHz) for 3 min. The cell suspension was filtered through a 0.22 μm × 50 mm sterile nylon membrane to separate the microbial cells from the leaves. The leaf DNA was directly extracted from each collected membrane.

Total DNA was extracted using the MoBio PowerSoil DNA Isolation Kit (MP Biomedicals, Santa Ana, CA, USA) from OMEGA, USA, and approximately 0.5 g of sample was weighed for each sample according to the extraction procedure specified by the kit. A NanoDrop ND-1000 spectrophotometer (Thermo Fisher Scientific, Waltham, MA, USA) was used to determine the quantity and quality of the extracted DNA. Primers 338F (5′-ACTCCTACGGGAGGCAGCA-3′) and 806R (5′-GGACTACHVGGGTWTCTAAT-3′) were used to amplify the V3–V4 region of the bacterial 16S rRNA gene [41]. The fungal ITS region was amplified with the primers ITS5 (5′-GGAAGTAAAAGTCGTAACAAGG-3′) and ITS2 (5′-GCTGCGTTCTTCATCGATGC-3′) [42]. The PCR amplification system had a total volume of 25 μL, which included 2 μL of DNA template, 0.4 μM each of the upstream and downstream primers (0.1 μL, 10 μmol·L^−1^), 5 μL of buffer, 5 μL of Q5 high-fidelity buffer, 0.25 μL of high-fidelity DNA polymerase, 0.2 mmol·L^−1^ dNTP (2.5 mmol·L^−1^, 2 μL), and 8.75 μL of ultrapure water (dd H_2_O). The PCR (ABI-2720 PCR instrument from the United States) amplification conditions consisted of first-predenaturing at 98 °C for 2 min, then repeated 25 times in a cycle of 98 °C for 15 s, 55 °C for 30 s, and 72 °C for 30 s, and with a final extension at 72 °C for 5 min. The PCR amplicons were purified by using Agincourt AM-Pure Beads (Beckman Coulter, Indianapolis, IN, USA), and quantified by using a Pico Green dsDNA detection kit (Invitrogen, Carlsbad, CA, USA). The PCR products were sequenced by using the Illumina NovaSeq 6000 sequencing platform at the Shanghai Personal Biotechnology Co., Ltd., Shanghai, China.

### 2.5. Statistical Analysis

Excel (2019) was used for data processing, and SPSS (26.0) was used for statistical analyses. The data in the table represent the repeated averages ± standard deviations. One-way ANOVA was used to analyze the differences in chemical properties of different litter leaves. Ecologists use alpha diversity and beta diversity indices to characterize the diversity of species within and between habitats, respectively, to comprehensively evaluate their overall diversity [43,44]. Data normalization was performed during the alpha diversity analysis. The leveling rule is to use the qiime feature-table rarefy function, and the leveling depth is set to 95% of the minimum sample sequence size. The amount of flattened data were 61,519. To comprehensively assess the alpha diversities of the microbial communities, Chao1 [45] and Observed-species indices were used to characterize the richness, and the Shannon [46,47] and Simpson [48] indices were used to characterize the diversity. The evenness was characterized by using Pielou’s evenness index [49], and the coverage was characterized by using Good’s coverage index [50]. The ggplot2 package in R (R v.3.4.4) was used to draw the boxplots. Principal coordinates analysis (PCoA) is one of the classic unconstrained sorting (classical multidimensional scaling, cMDScale) analysis methods [51]. According to the OTU (Operational Taxonomic Units) table and ape package in R (R v.3.4.4), the differences in the β-diversity of litter leaves were analyzed and compared. Among the samples, the shared and unique OTUs of the leaf microbial communities were analyzed in R (R v.3.4.4), and the “Venn Diagram” package was used to create Venn diagrams. Heatmaps for the top 50 taxonomic genera in each sample were constructed using R (R v.3.4.4) and the pheatmap package. The linear discriminant analysis (LDA) effect size (LEfSe) method was used to detect potentially biomarker-rich taxa based on a cross-group normalized relative abundance matrix using default parameters. Its essence is to combine linear discriminant analysis with nonparametric Kruskal–Wallis and Wilcoxon rank sum tests to screen for key biomarkers (e.g., key community members) [52]. The matrix was constructed using Galaxy, which conducts an online interactive analysis of microbial community data. For studies of microbial ecology, the functional potential of the flora is also worthy of attention. Microbial function prediction data analysis was implemented through R (R v.3.4.4).

## 3. Results

### 3.1. Chemical Properties of Leaves with Different Proportions of Leaf Litter

Carbon (C) is the most basic structural element in plants, while nitrogen (N) and phosphorus (P) are both essential functional elements for plant growth and development and are the common limiting elements [53]. The interactions among the three regulate plant growth [54,55]. Table 1 shows that there were significant differences in the total C, total N, and total P contents of different proportions of litter (*p* < 0.01). The total C and total N contents of PsMa were lower than those of Ps and Ma. C/N had the highest Ma, followed by Ps, and PsMa, while both N/P and C/P exhibited the highest Ps and lowest Ma values. It is worth noting that the C/N differences in the proportions of the three litter species were not significant (*p* > 0.05) (Table 1).

### 3.2. Microbial Community Compositions and Structural Characteristics of Different Leaf Litter Ratios

**Figure 2:**
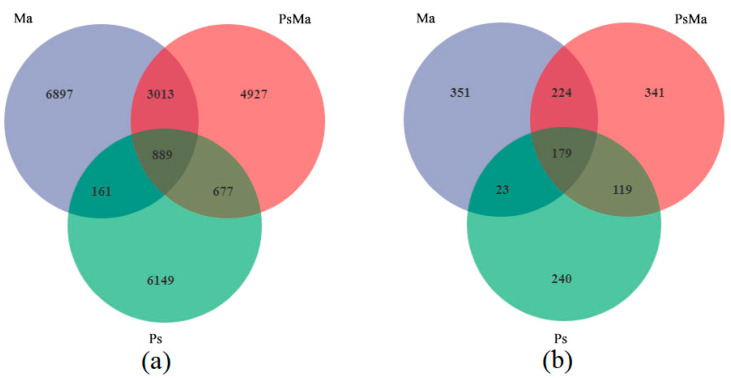
Venn diagram showing unique and shared OTUs of leaf litter microbial for three different samples. (**a**): Unique and shared OTUs of leaf litter bacteria in three different samples; (**b**): Unique and shared OTUs of leaf litter fungal in three different samples. Ma: *Morus alba*; PsMa: *Pinus sylvestris* var. *mongolica* × *Morus alba*; Ps: *Pinus sylvestris* var. *mongolica*.

**Figure 3:**
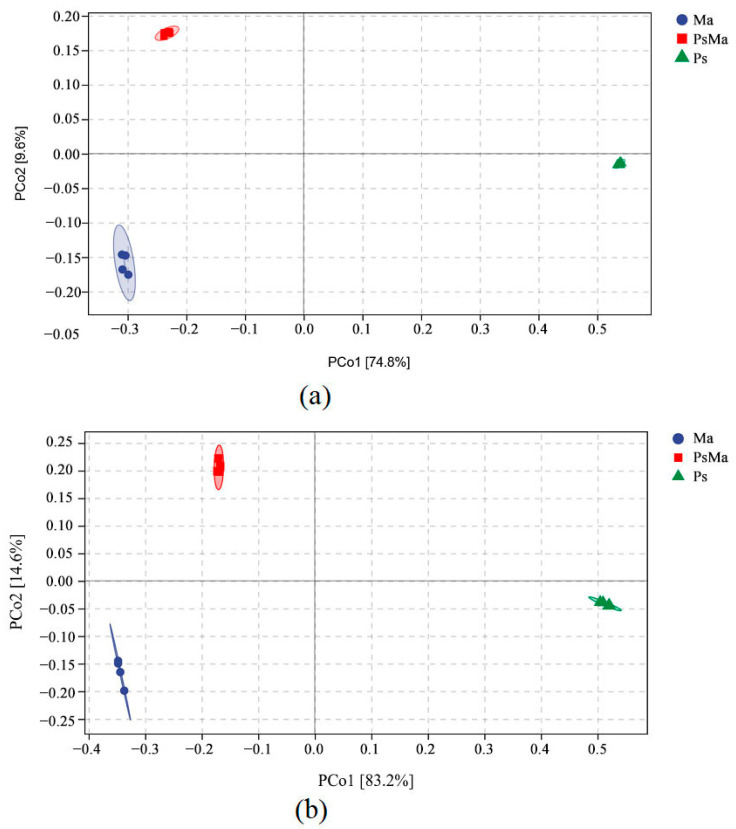
Principle coordinate analysis (PCoA) of Bray–Curtis’s distance from all samples. (**a**): PCoA of bacterial communities in different samples; (**b**): PCoA of fungal communities in different samples. Ma: *Morus alba*; PsMa: *Pinus sylvestris* var. *mongolica* × *Morus alba*; Ps: *Pinus sylvestris* var. *mongolica*. The circle in the figure is the 95% confidence ellipse.

At the bacterial level, a total of 22,713 OTUs were aggregated. Ma, PsMa and Ps had 10,969, 9506 and 7866 OTUs, respectively. The number of OTUs that were shared by Ma, PsMa, and Ps was 889, and the unique OTUs of Ma, PsMa, and Ps were 6897, 4927, and 6149, respectively (Figure 2a). At the fungal level, a total of 1477 OTUs were aggregated. Ma, PsMa and Ps had 777, 863, and 561 OTUs, respectively. The number of OTUs that were shared by Ma, PsMa, and Ps was 179, and the numbers of unique OTUs that were shared by Ma, PsMa, and Ps were 351, 341, and 240, respectively (Figure 2b). Unconstrained principal coordinate analysis (PCoA) using Bray–Curtis distances revealed that the compositions of the litter bacterial and fungal communities all differed among Ma, PsMa, and Ps and formed three distinct clusters that were separated along the first coordinate axis (Figure 3).

**Figure 4:**
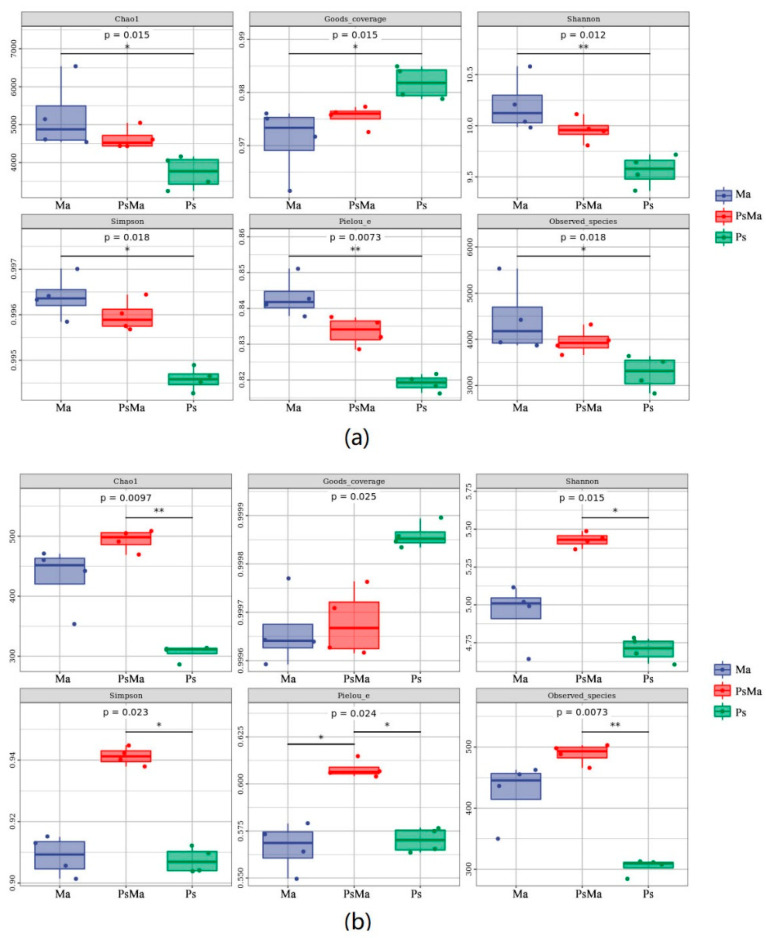
Litter microbial diversity index in Ma, PsMa and Ps. (**a**): Alpha diversity analysis of leaf litter bacterial community; (**b**): Alpha diversity analysis of leaf litter fungal community. Ma: *Morus alba*; PsMa: *Pinus sylvestris* var. *mongolica* × *Morus alba*; Ps: *Pinus sylvestris* var. *mongolica*. ** meant significant difference at 0.01 level. * meant significant difference at 0.05 level.

Alpha diversity index analysis was performed on samples with different leaf litter ratios, and boxplots were drawn. The litter bacterial diversity indices, including the Chao1 index (*p* = 0.015), Pielou_e index (*p* = 0.0073), Goods_coverage (*p* = 0.015), Shannon index (*p* = 0.012), Simpson index (*p* = 0.018) and Observed_species (*p* = 0.018), showed significant differences among Ma, PsMa and Ps. Ma had the highest Chao1 index, Pielou_e index, Shannon index, Simpson index and Observed_species, which were 5209.033, 0.843, 10.204, 0.996 and 4439.675, respectively, followed by PsMa, while Ps had the lowest. Ma had the highest abundance, diversity and evenness (Figure 4a). However, the fungal results were different from that obtained with bacteria. Litter bacterial diversity index, including Chao1 index (*p* = 0.0097), Pielou_e index (*p* = 0.024), Shannon index (*p* = 0.015), Simpson index (*p* = 0.023) and Observed_species (*p* = 0.0073), exhibited significant differences among Ma, PsMa and Ps. Ps had the highest Chao1 index, Pielou_e index, Shannon index, Simpson index and Observed_species, which were 493.582, 0.608, 5.430, 0.941 and 488.7, respectively, were followed by Ma, and Ps had the lowest. The Goods_coverage index showed the opposite pattern, namely, Ps > PsMa > Ma. PsMa exhibited the highest abundance, diversity and evenness (Figure 4b).

**Figure 5:**
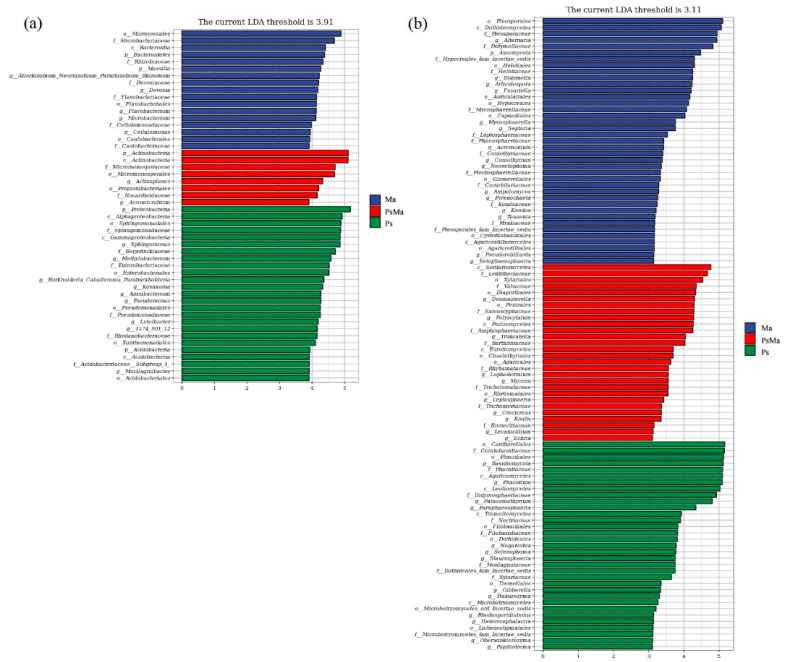
Microbial community of different leaf litter ratios with significantly different taxa. (**a**): litter bacterial communities; (**b**): litter fungal communities. Ma: *Morus alba*; PsMa: *Pinus sylvestris* var. *mongolica* × *Morus alba*; Ps: *Pinus sylvestris* var. *mongolica*. In the significantly changed bacterial taxon under different litter types, the ordinate is a taxonomic unit with significant differences between groups, and the abscissa visualizes the logarithmic scores of the LDA difference analysis corresponding to the taxon and sorts them according to the size of the scores to describe them as different. The size of the difference in the grouped sample. The longer the length, the more significant the difference between the taxon units, and the different color of the bar chart indicates the higher abundance sample group corresponding to the taxon.

**Figure 6:**
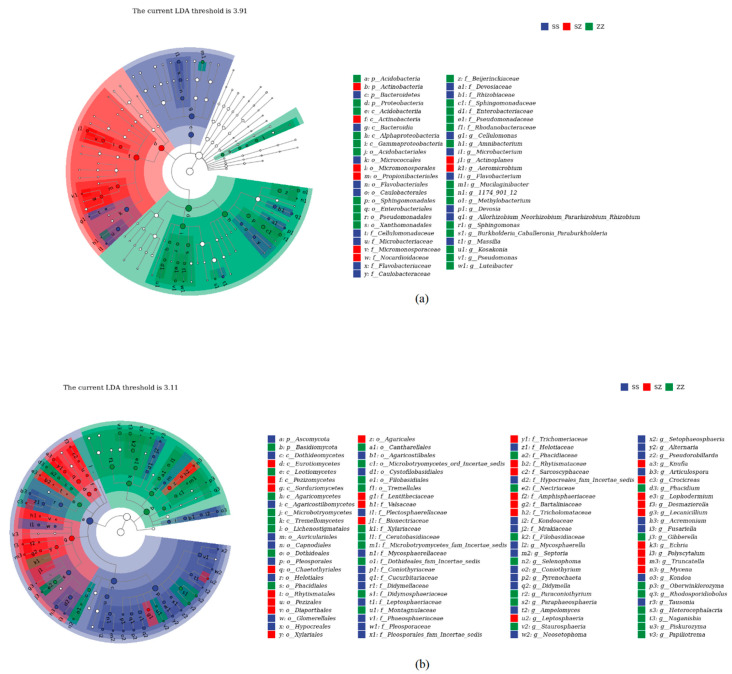
(**a**): Lefse with an LDA of 3.91 indicates that a significantly difference between litter bacterial communities of Ma, PsMa, and Ps. (**b**): Lefse with an LDA of 3.11 indicates a significantly difference between litter fungal communities of Ma, PsMa, and Ps. Ma: *Morus alba*; PsMa: *Pinus sylvestris* var. *mongolica* × *Morus alba*; Ps: *Pinus sylvestris* var. *mongolica*. The taxonomic cladogram shows the taxonomic hierarchies of the main taxa from phylum to genus (from inner circle to outer circle) in the sample community. Node size corresponds to the average relative abundance of that taxon; hollow nodes represent taxa that are not significantly different between groups, while nodes in other colors (e.g., green and red) indicate that these taxa exhibit significant between-group differences, and the abundance is higher in the grouped samples represented by this color. Letters identify the names of taxa that differ significantly between groups.

With LDA effect size scores >4.5, 16 bacterial taxa were significantly different across treatments (Figure 5a). When the LDA effect size scores were >5, 3 bacterial taxa were significantly different in the litter from PsMa and Ps. Among them, at the phylum level, the main enriched bacterial taxa in the Ma leaf litter were Bacteroidetes, PsMa was mainly enriched by Actinobacteria, and Ps were enriched by Proteobacteria (Figure 6a). As shown in Figure 5b, when the LDA effect size scores were greater than 4, the relative abundances of 40 fungal taxa were significantly different among the different treatments (*p* < 0.05). At the phylum level, Ascomycota was mainly enriched in the Ma and PsMa litters, while Basidiomycota was mainly enriched in Ps (Figure 6b).

**Figure 7:**
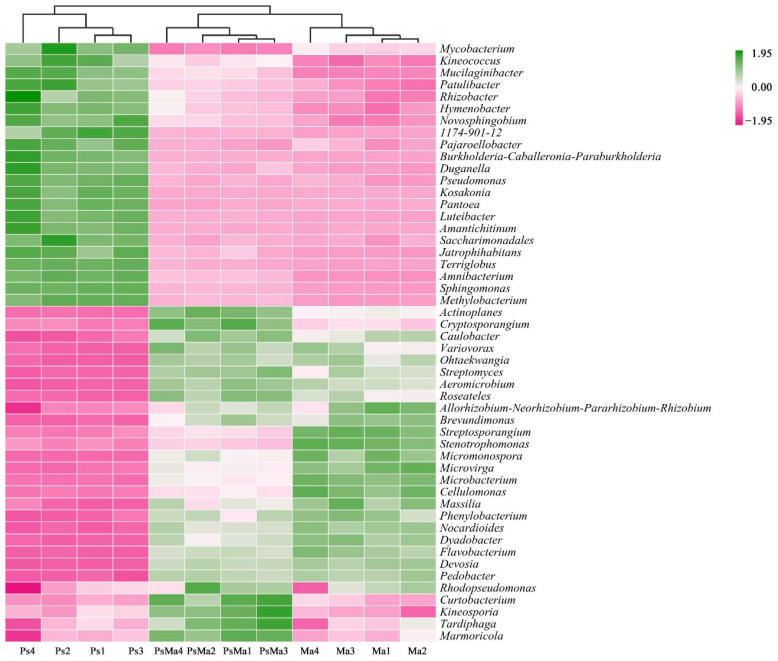
Heatmap and cluster analysis based on relative abundance of the top 50 genera identified in litter bacterial communities. The samples are grouped according to their similarity to each other. In the figure, pink represents the genus with lower abundance in the corresponding sample, green represents the genus with higher abundance, and the color change represents the level of abundance. Ma: *Morus alba*; PsMa: *Pinus sylvestris* var. *mongolica* × *Morus alba*; Ps: *Pinus sylvestris* var. *mongolica*.

**Figure 8:**
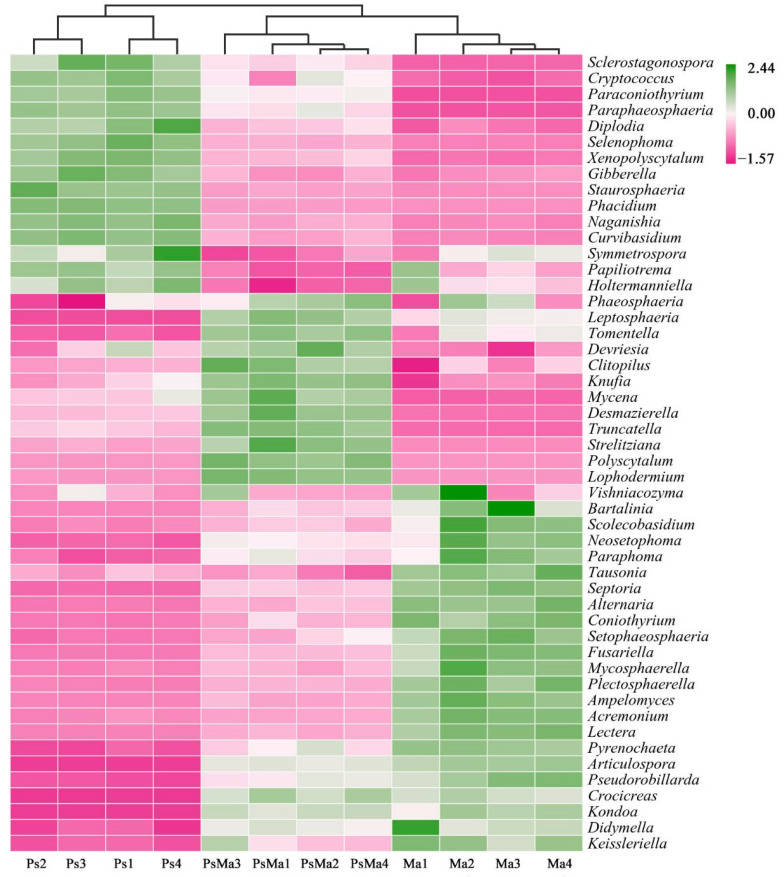
Heatmap and cluster analysis based on relative abundance of the top 50 genera identified in litter fungal communities. The samples are grouped according to their similarity to each other. In the figure, pink represents the genus with lower abundance in the corresponding sample, green represents the genus with higher abundance, and the color change represents the level of abundance. Ma: *Morus alba*; PsMa: *Pinus sylvestris* var. *mongolica* × *Morus alba*; Ps: *Pinus sylvestris* var. *mongolica*.

The Bray–Curtis-based heatmap showed that the litter bacterial communities of Ma and PsMa were clustered together, which indicated that the litter leaf communities from Ps were clearly distinct from those of Ma and PsMa (Figure 7). The fungal communities of the litters also exhibited the same properties (Figure 8).

### 3.3. Prediction of Microbial Community Functions with Different Leaf Litter Ratios

**Figure 9:**
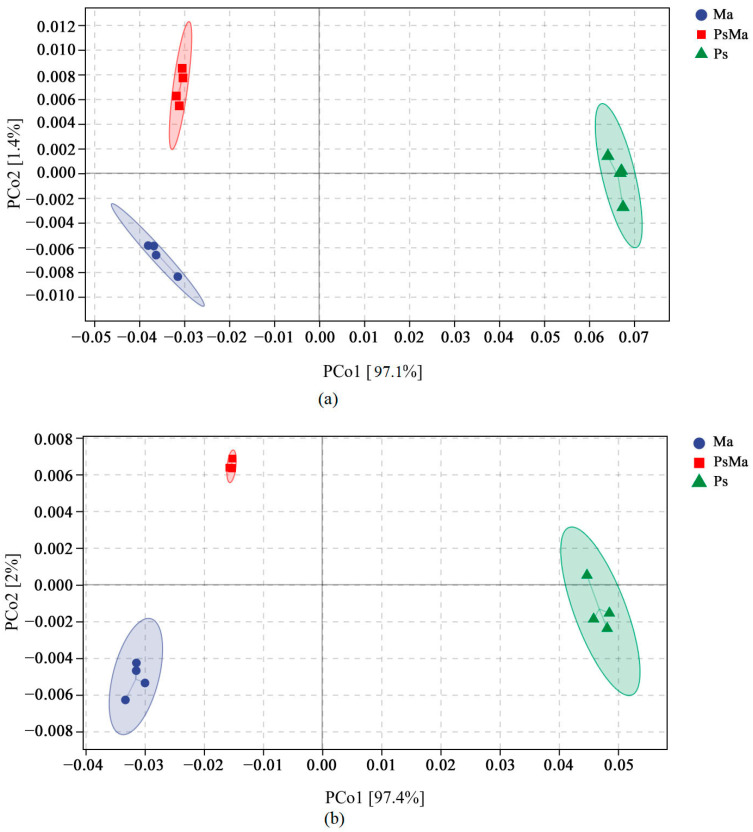
Functional unit PCoA analysis of different litters. (**a**): Functional unit PCoA analysis of bacterial communities with different leaf litter ratios; (**b**): Functional unit PCoA analysis of fungal communities with different leaf litter ratios. Ma: *Morus alba*; PsMa: *Pinus sylvestris* var. *mongolica* × *Morus alba*; Ps: *Pinus sylvestris* var. *mongolica*. The circle in the figure is the 95% confidence ellipse.

The sample difference distance matrix (Bray–Curtis’s distance is used by default) was combined with principal coordinate analysis to expand the sample functional differences in two dimensions and provided the principal coordinate analysis map of the microbial functional units of the different litter types. As shown in Figure 9a, the first two axes of functional units of the different litter bacterial communities accounted for 98.5% of the total variance (e.g., PCo1 97.1% and PCo2 1.4%). The first two axes of the fungal communities accounted for 99.4% (e.g., PCo1 97.4% and PCo2 2%). For the bacterial and fungal communities, Ma, PsMa, and Ps were clearly separated along the PCo1 axis. Ma and PsMa were located on the negative semiaxis of PCo1, and Ps was located on the positive semiaxis (Figure 9b).

**Figure 10:**
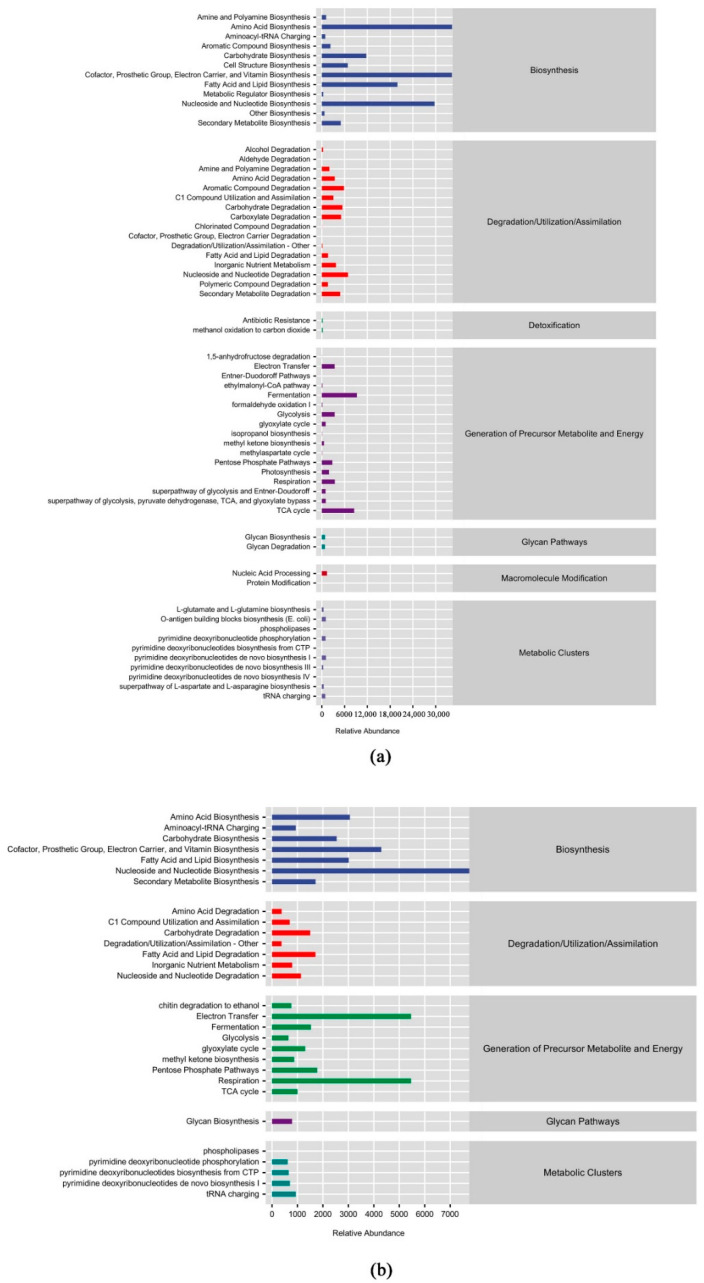
Metabolic pathway statistics of microbial communities with different leaf litter ratios. (**a**): litter bacterial communities; (**b**): litter fungal bacterial.

Figure 10 mainly focused on the second-level pathway analysis. The common functions that were predicted by the bacterial and fungal communities were biosynthesis, degradation/utilization/assimilation, generation of precursor metabolite and energy, glycan pathways, and metabolic clusters; in addition, the bacterial communities were predicted to have detoxification and macromolecule modification functions. In terms of biosynthesis, the fungal communities were functionally relatively abundant in biosynthesis, degradation/utilization/assimilation, and generation of precursor metabolite and energy, while the bacterial communities were functionally relatively abundant only in biosynthesis. The relative abundances of fungal communities were significantly higher than those of bacterial communities in terms of the precursor metabolites and energy production functions.

**Figure 11:**
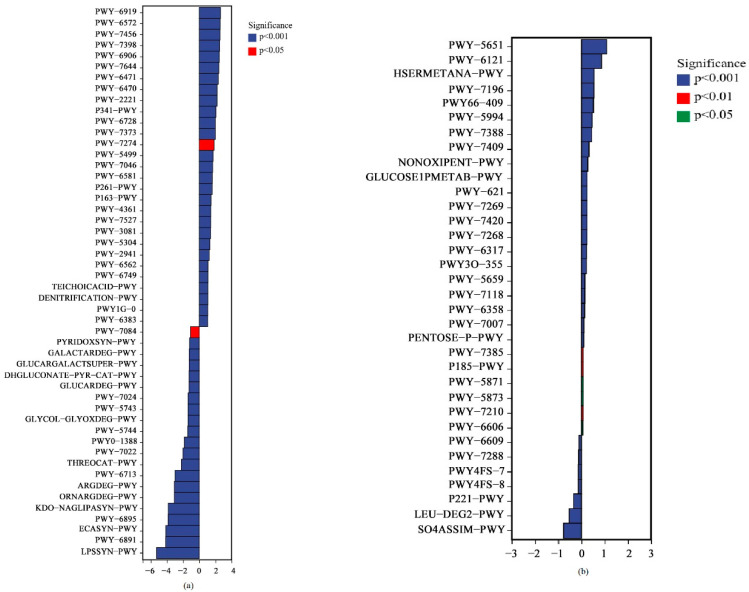
Differential analysis of metabolic pathways in microbial communities between Ma, PsMa, and Ps. (**a**): litter bacterial communities; (**b**): litter fungal bacterial. Ma: *Morus alba*; PsMa: *Pinus sylvestris* var. *mongolica* × *Morus alba*; Ps: *Pinus sylvestris* var. *mongolica*.

After obtaining the abundance data of the metabolic pathways, we used Ps as the control group and PsMa as the upregulated group and attempted to determine the metabolic pathways with significant differences among the groups. As shown in Figure 11a, in the bacterial communities, except for PWY-7274 and PWY-7084 (*p* < 0.05), the rest of the metabolic pathways were significantly different in different leaf litters (*p* < 0.001). Among the fungal communities of different litter leaves, PWY-7210, PWY-7385, and P185-PWY were not significant (*p* < 0.01). PWY-6606, PWY-5873 and PWY-5871 had significant differences (*p* < 0.05), and the rest had extremely significant differences (*p* < 0.001) (Figure 11b).

## 4. Discussion

As one of the important components of forest ecosystems, although litter accounts for a small proportion of the total forest biomass, it not only affects forest biomass but also plays an important role in total forest productivity, material cycling, and nutrient return. This is because the turnover rates of nutrient litter elements are faster than those in trees, which rely on their own metabolisms to absorb and transform nutrients [56]. Most studies also have indicated that the mixed decomposition of coniferous and broadleaf trees can significantly promote the decomposition of coniferous litter and its nutrient releases [57,58]. The main reason may be that when coniferous and broad-leaved litter are mixed and decomposed, the higher-quality broad-leaved litter will provide nutrients for the lower-quality coniferous litter, which thereby eases the nutrient limitations on microorganisms during the decomposition process [59,60].

Plant litter quality is the main factor that affects nutrient release, and its C/N value is often regarded as an important attribute in measuring litter quality [61,62]. For example, Brady and Weil [63] found that nitrogen fixation occurs when the C/N ratios in the remaining litter are greater than 25, and nitrogen release occurs when C/N < 25 [64]. In this study, the C/N values of the three litter types were all <25, which were in the nitrogen release state. The release of nitrogen from the coniferous litter was slow, while the release of nitrogen from mixed coniferous and broad-leaved litter was significantly accelerated. Early litter decomposition C/N can also control the decomposition rate [65,66]. Low C/N is conducive to the release of nutrients by microorganisms in the organic matter decomposition process [67]. In this study, it was found that the C/N of PsMa was lower than that of Ps, which indicated that the nutrient releases by microorganisms in the mixed forest during the decomposition of organic matter were better than those in the pure *P. sylvestris* var. *mongolica* forest. The litter decomposition rate affects the nutrient cycling of forest ecosystems [68]. Studies have shown that the C/N and C/P of litter can characterize the litter decomposition rates, and the higher the C/N and C/P are, the lower the decomposition rate [69,70]. Chen et al. [71] believed that when the litter C/N > 27 or C/P > 186, the decomposition of litter will be inhibited. Liu et al. [72] and Pan et al. [73] both found that litter N/P can be used as an indicator for judging nutrient limitations. If the litter N/P > 25, then litter decomposition is limited by P. It can be seen that in this study, the addition of *M. alba* leaf litter increased the decomposition rate of *P. sylvestris* var. *mongolica* litter. In summary, it can be inferred that the litter decomposition rates of the three proportions used in this study were different and reflected that mixing of *M. alba* and *P. sylvestris* var. *mongolica* could effectively improve the decomposition of litter in pure *P. sylvestris* var. *mongolica* stands. In summary, it can be inferred that the litter decomposition rates of the three proportions used in this study were different, and, as seen from the N/P values, the mixing of *M. alba* and *P. sylvestris* var. *mongolica* could effectively improve litter decomposition in pure *P. sylvestris* var. *mongolica* stands.

At present, there have been many reports on the decomposition of mixed litter. The microbial community structure of mixed litter is significantly different from those of litter from single tree species, which has also been confirmed in previous studies [74,75,76,77]. This is because differences in litter chemical compositions or microenvironments can lead to differences in microbial biomass and community compositions [78,79,80,81,82]. With an increase in the proportion of *M. alba* leaf litter, the bacterial community Chao1 index and Simpson and Shannon indices decreased. However, the present study found that pure *M. alba* forest and *M. alba* × *P. sylvestris* mixed forest had no significant effect on the α-diversity of the leaf fungal community (Chao1, Shannon, Simpson) (*p* > 0.05), which was consistent with previous research results [83,84]. The main reason may be that the heterogeneity of leaf litter resources provides different nutrients and living environments for the growth of leaf microorganisms, which leads to different fungal community diversity indices. The study found that in the early stage of litter decomposition, Proteobacteria and Ascomycetes were the most abundant taxa and were the main decomposers, which was consistent with previous research conclusions [85,86]. Mixed forest litter alters the litter carbon and mineral nutrient contents compared to pure coniferous forest litter, which thereby provides a broader substrate for decomposing microorganisms [87,88]. Therefore, compared with pure coniferous forest litter, mixed forest litter can significantly improve the richness and diversity of microbial communities and can ameliorate the decline of pure coniferous forest.

Microbial community structures and metabolic functions are closely related [89]. Biosynthesis, degradation/utilization/assimilation, production of precursor metabolites and energy, glycan pathways, and metabolic clusters were the common predicted functions of the microbial communities examined in this study. In addition, bacteria were predicted to have detoxification and macromolecular modification functions. In terms of biosynthesis, fungi were relatively functionally rich in biosynthesis, degradation/utilization/assimilation, and production of precursor metabolites and energy, while bacteria were relatively functionally rich only in biosynthesis. Differences in litter chemical composition or litter quality often lead to differences in microbial community compositions and functions. For example, with the improvement in litter quality, the numbers of saprophytic fungi increased, the numbers of ammonia-oxidizing bacteria decreased, and the microbial community changes that were mediated by the litter quality affected ecosystem functions [90]. Many studies have found that Proteobacteria, Actinobacteria, and Ascomycota are the most abundant phyla in the early stage of litter decomposition, and they are considered to be the main decomposers [91]. In this study, Actinobacteria and Proteobacteria dominated the bacterial community, and Ascomycota dominated the fungal community. Proteobacteria are eutrophic bacteria that lead to faster nutrient returns from litter leaves, which results in improved soil nutrient availability. From the perspective of trophic type, the saprophytic trophic type is the most important trophic type, which may be related to Ascomycota being the most dominant phylum. Ascomycota are mostly saprophytic fungi and are important decomposers that can decompose refractory organic matter and play an important role in nutrient cycling [92]. Fungi are primarily responsible for the decomposition of carbonaceous organic matter [93,94,95], while bacteria primarily utilize nitrogenous organic matter [96]. This study shows that fungi are rich in carbohydrate metabolism genes, while bacteria are rich in amino acid metabolism genes, which may be due to the predominance of Ascomycetes, which are the decomposing bacteria in the three litter types and are mainly responsible for decomposing cellulose and hemicellulose [97,98,99]. Proteobacteria are mainly responsible for breaking down proteins and amino acids [100,101].

## 5. Conclusions

In this study, a mixed forest composed of *Pinus sylvestris* var. *mongolica* and *Morus alba* was simulated by the mixed mode of litter under a *P. sylvestris* var. *mongolica* forest, and the litter physicochemical properties, microbial structures and communities were analyzed. It was found that the mixed litter of *P. sylvestris* var. *mongolica* and *M. alba* can significantly improve the microbial structure and community diversity of pure pine forest litter. This study provides a basis for exploring the relationships among forest trees to provide guidance for the introduction of tree species with coordinated interspecific relationships and for establishing mixed forests in pure *P. sylvestris* var. *mongolica* forests.

## Figures and Tables

**Table 1:** Chemical properties of leaves with different proportions of leaf litter.

Different Samples	Total C/g·kg^−1^	Total N/g·kg^−1^	Total P/g·kg^−1^	C/N	N/P	C/P
Ps	824.50 ± 12.87 bB	54.00 ± 0.82 bB	0.78 ± 0.15 cB	15.27 ± 0.10 aA	71.75 ± 14.37 aA	1094.78 ± 214.60 aA
Ma	923.50 ± 43.80 aA	58.00 ± 0.82 aA	3.73 ± 0.59 aA	15.93 ± 0.81 aA	15.87 ± 2.75 cB	253.62 ± 50.76 cB
PsMa	731.00 ± 25.53 cC	49.25 ± 1.26 cC	1.51 ± 0.49 bB	14.85 ± 0.57 aA	35.32 ± 11.07 bB	525.95 ± 168.83 bB
F test	40.64	78.94	46.18	3.60	28.68	28.66

Different small letters meant significant difference at 0.05 level. Different capital letters meant significant difference at 0.01 level. Ma: *Morus alba*; PsMa: *Pinus sylvestris* var. *mongolica* × *Morus alba*; Ps: *Pinus sylvestris* var. *mongolica*.

## Data Availability

The data presented in this study are available on request from the corresponding author. The data are not publicly available due to policy of the institute.

## Supplementary Materials

The following supporting information can be downloaded at: https://www.mdpi.com/article/10.3390/microorganisms10061117/s1, Figure S1: Litter Collector.
