# Supplementary material for: Effects of Mixed Decomposition of Pinus sylvestris var. mongolica and Morus alba Litter on Microbial Diversity
Source: Microorganisms. 2022 May 28;10(6):1117. doi: 10.3390/microorganisms10061117 (PMC9229243; doi:10.3390/microorganisms10061117)
Supplement: Supplementary file 1 [file microorganisms-10-01117-s001.zip › microorganisms-1700166-supplementary.pdf]

Supplementary Material

Effects of mixed decomposition of *Pinus sylvestris* var. *mongolica* and *Morus alba* litter on microbial diversity

Jiaying Liu [1,3], Yawei Wei [1,2], You Yin [1], Keye Zhu [1], Yuting Liu [1], Hui Ding [1], Jiawei Lei [1], Wenxu Zhu [1,2]* and Yongbin Zhou [3,4*]

[1] College of Forestry, Shenyang Agricultural University, Shenyang, China; ljying222@163.com (J.L.); 2013500010@syau.edu.cn (Y.W.); 1993500012@syau.edu.cn (Y.Y.); ansizky@163.com (K.Z.); lyt_edu@163.com (Y.L.); a15502410421@163.com (H.D.); leijiwei123456@163.com (W.L.); zhuwx@syau.edu.cn (W.Z.)
[2] Research Station of Liaohe-River Plain Forest Ecosystem, Chinese Forest Ecosystem Research Network (CFERN), Shenyang Agricultural University, Tieling, China; 2013500010@syau.edu.cn (Y.W.); zhuwx@syau.edu.cn (W.Z.)
[3] Institute of Modern Agricultural Research, Dalian University, Dalian, China; ljying222@163.com (J.L.); 1999500010@syau.edu.cn (Y.Z.)
[4] Life Science and Technology College, Dalian University, Dalian, China; 1999500010@syau.edu.cn (Y.Z.)
* Correspondence: zhuwx@syau.edu.cn; 1999500010@syau.edu.cn Tel.: (86-13998160246)

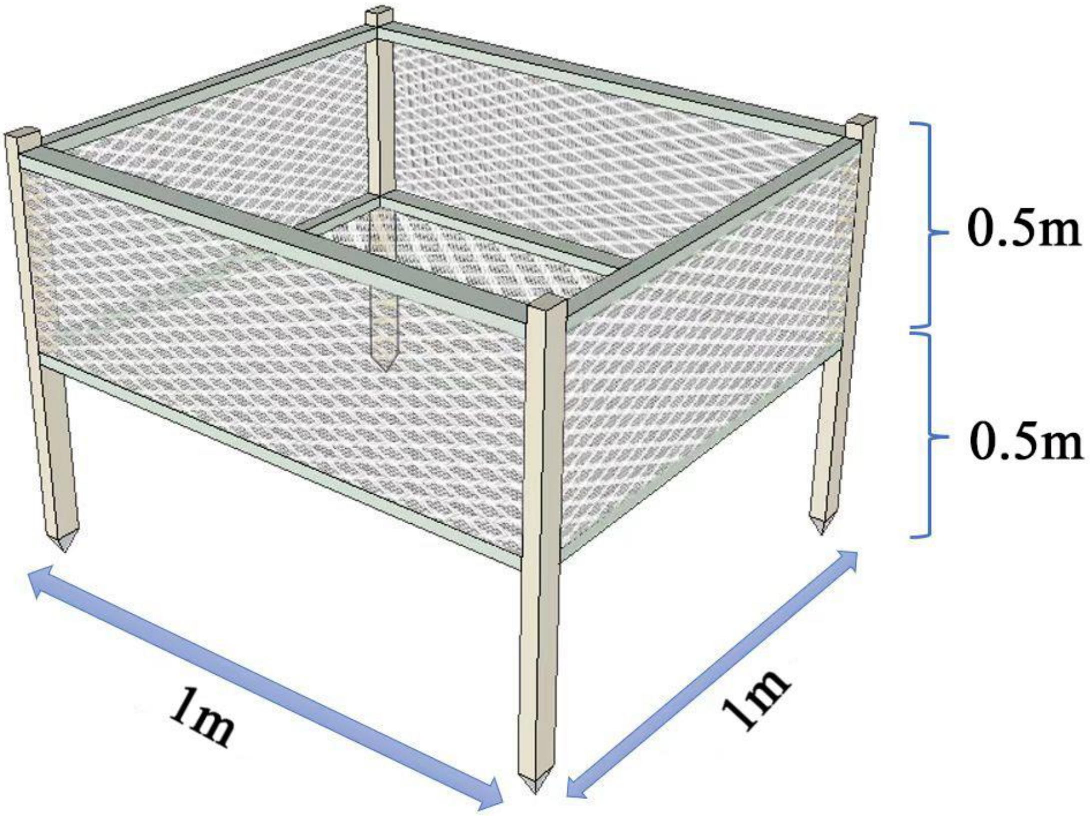

Figure S1. Litter collector. The size of the litter collector is 1m x 1m.
